# Anti-B Cell Strategy in Nephrotic Syndrome: Beyond Rituximab

**DOI:** 10.3390/biomedicines13092063

**Published:** 2025-08-24

**Authors:** Yanyan Jin, Yi Xie, Haidong Fu, Fei Liu, Jianhua Mao

**Affiliations:** Department of Nephrology, The Children’s Hospital, Zhejiang University School of Medicine, National Clinical Research Center for Child Health, Hangzhou 310052, China

**Keywords:** NS, rituximab, B cell-targeted therapies, CD38 monoclonal antibodies, CAR T cell therapy

## Abstract

Nephrotic syndrome (NS) is a complex kidney disorder characterized by profound proteinuria, hypoalbuminemia, hyperlipidemia, and edema, significantly impacting patients’ quality of life. While corticosteroids and calcineurin inhibitors (CNIs) have traditionally been the primary treatments, B cell-targeted therapies, especially the anti-CD20 monoclonal antibody rituximab, have transformed the management of steroid-dependent and multidrug-resistant NS (MRNS). Rituximab has demonstrated efficacy in reducing relapse rates and steroid dependence by depleting CD20+ B cells, which play a pivotal role in autoantibody production and immune dysregulation. However, limitations such as incomplete B cell depletion, immunogenicity leading to anti-rituximab antibodies, and variable efficacy in refractory cases have led to the development of next-generation therapies. This review critically examines recent advances in B cell-targeted therapies for NS, with a particular focus on overcoming the limitations of conventional rituximab treatment. This review systematically analyzes next-generation anti-CD20 monoclonal antibodies, CD38-targeted therapies, and emerging CAR-T cell approaches, evaluating their distinct mechanisms of action and clinical trial outcomes. The analysis extends to innovative combination strategies and biomarker-guided treatment algorithms for refractory cases. By synthesizing preclinical data with clinical evidence, this work provides a framework for optimizing therapeutic decision-making in NS, while identifying key knowledge gaps that warrant future investigation. Collaborative research and translational studies are essential for advancing precision medicine in NS, ensuring that new therapies provide lasting clinical benefits for patients. The evolving field of anti-B cell therapies marks a new era in managing refractory NS, offering hope for better long-term prognoses.

## 1. Introduction

In the field of nephrology, the anti-B cell strategy, especially the utilization of monoclonal antibodies, has emerged as a crucial therapeutic modality for the management of NS, as demonstrated by the efficacy in treating membranous nephropathy (MN). This syndrome, characterized by its complexity and multifactorial nature, is distinguished by a spectrum of underlying pathologies that collectively result in the increased permeability of the glomerular basement membrane. This pathological alteration precipitates a significant excretion of substantial quantities of plasma proteins into the urine, a phenomenon that holds both diagnostic and prognostic implications for the syndrome.

NS exhibits remarkably diverse clinical manifestations, profoundly affecting a patient’s quality of life. The defining feature of NS, massive proteinuria, is often accompanied by hypoalbuminemia, a condition marked by reduced levels of albumin in the blood. This deficiency exacerbates the patient’s condition, leading to hyperlipidemia, an abnormal elevation in lipid levels, and significant edema, which manifests as the visible swelling of tissues due to fluid retention [1].

**Figure 1:**
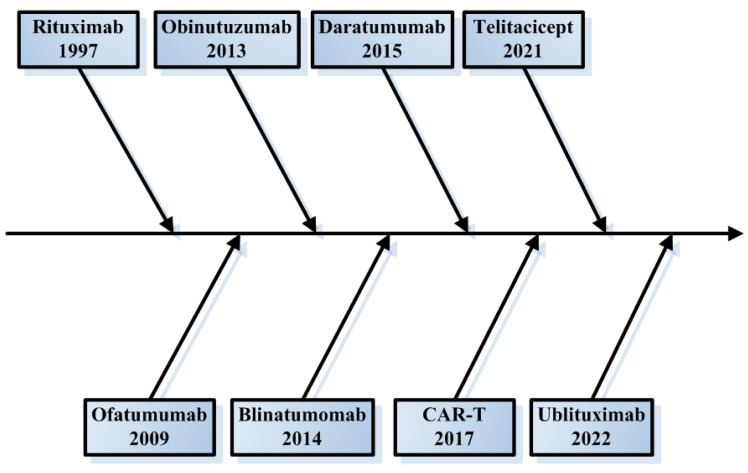
Chronology of pertinent therapeutic advancements.

It is widely acknowledged that glucocorticoids are linked to a broad spectrum of adverse effects which restrict their efficacy in long-term therapeutic applications. A specific group of patients experiencing frequent relapses does not attain a reduction in glucocorticoid dosage, even after undergoing immunosuppressive therapy. The detrimental effects of immunosuppressants, including myelosuppression, nephrotoxicity, and gastrointestinal/cardiovascular complications, are increasingly acknowledged, with the prolonged use of these agents further escalating the risk of malignancies. Although the ongoing use of corticosteroids and CNIs as primary treatments, B cell-targeted approaches, notably the use of rituximab, a chimeric anti-CD20 monoclonal antibody, have markedly altered the therapeutic paradigm for steroid-dependent NS (SDNS) and MRNS [2,3]. Nevertheless, obstacles such as incomplete B cell depletion, immunogenicity, and recurrence have propelled the development of innovative anti-B cell therapeutic strategies [4]. This review explores a systematic analysis of next-generation anti-CD20 monoclonal antibodies and innovative approaches such as chimeric antigen receptor (CAR)-T cell therapy, focusing on their distinct mechanisms of action, clinical efficacy, and safety profiles derived from recent trial data (Figure 1, Table 1). By integrating current evidence with mechanistic insights, this work aims to inform clinical decision-making and guide future therapeutic development in NS.

## 2. The Role of B Cells and Autoantibodies in NS

**Figure 2:**
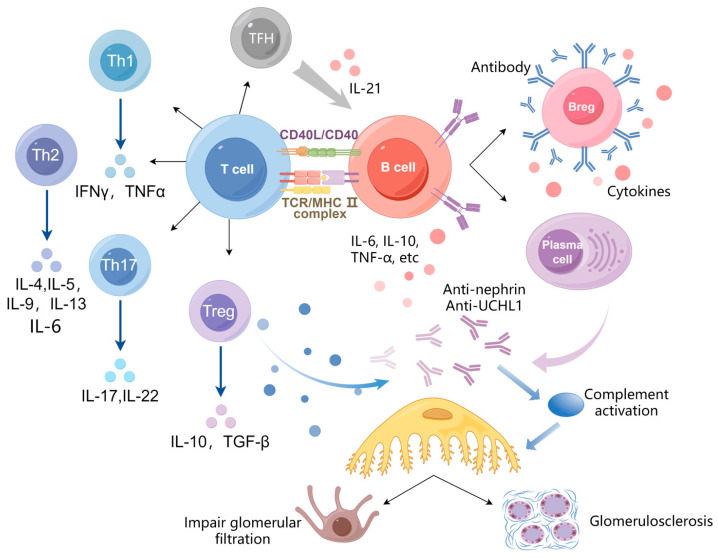
The possible pathological mechanism of B cells in NS: B cells drive NS pathogenesis through four key mechanisms: autoantibody-mediated podocyte injury (anti-nephrin, anti-UCHL1), immune complex formation and complement activation, proinflammatory cytokine production (IL-6, IL-10, TNF-α, etc.), and disruption of regulatory B cell function. These processes collectively impair glomerular filtration, promote proteinuria, and accelerate progression to glomerulosclerosis, with therapeutic response to B cell depletion therapies confirming their pathogenic role. The autoimmune and inflammatory effects of B cells ultimately lead to irreversible renal damage in susceptible patients. (Created in Figdraw (https://www.figdraw.com/). Authorization code ID: IYITYf9ff9).

The pathological mechanism of B cells in NS is a complex and multifaceted process (Figure 2). The following overview delineates the manner in which B cells contribute to the development of NS:

(1) Generating antibodies

B cells play a crucial role in the humoral immune response by producing antibodies [5,6]. Unlike typical antibodies that target external substances, autoantibodies are erroneously directed and attack the body’s own tissues [7]. In the case of NS, B cells may generate autoantibodies that specifically target the glomeruli, the microscopic filtering units within the kidneys [8,9]. These glomeruli play a vital role in filtering waste products and excess fluid from the bloodstream. When autoantibodies target the glomeruli, they inflict damage on the delicate filtration barrier, thereby increasing its permeability. This disruption permits proteins, which are meant to stay in the blood, to seep into the urine, ultimately causing the characteristic proteinuria associated with NS [9,10]. Hengel FE et al. and Watts AJB et al. conducted investigations into the role of antinephrin autoantibodies in podocytopathies. Their research indicated that antinephrin autoantibodies serve as indicators of disease activity and are likely to play a role in podocyte injury associated with minimal change disease (MCD) [6,9]. B cell depletion therapies, such as rituximab, have demonstrated efficacy, lending support to the notion of an autoimmune etiology in a subset of patients with MCD [6]. Jamin A et al. identified UCHL1 as a target cellular protein for a specific group of autoantibodies in patients experiencing relapses. Furthermore, their research offered additional evidence supporting the pathogenic role of anti-UCHL1 autoantibodies in the progression of idiopathic NS (INS) [7]. These autoantibodies can trigger an inflammatory response, causing glomerular damage and leading to the symptoms of NS, including proteinuria, hematuria, edema, and hypertension [8].

(2) Engage in the cellular immune response

B cells also participate in the cellular immune response through antigen presentation and cytokine production, which is essential in orchestrating and amplifying the inflammatory response. They participate in a complex interplay with other immune cells, including T cells and macrophages, to propagate inflammation [11,12].

In NS, the interplay between B cells and T cells plays a pivotal role in the onset and progression of the disease. As antigen-presenting cells, B cells engage with CD4+ T cells via MHC-II molecules and co-stimulatory signals, thereby facilitating the hyperactivation of effector T cell populations, including Th1, Th2, and Th17. A shift toward Th2 dominance results in elevated levels of IgE, while an imbalance between Th17 and Treg cells amplifies inflammatory processes [13]. Concurrently, activated T cells enhance B cell functions, such as proliferation, immunoglobulin class switching, and germinal center formation, through mechanisms like the CD40-CD40L pathway. This interaction leads to the generation of autoantibodies and pro-inflammatory cytokines, including IL-6 and IFN-γ [14]. Follicular helper T cells (TFH) promote disease progression by secreting IL-21, which further stimulates B cell differentiation into plasma cells and memory B cells, thereby establishing a vicious cycle [11,15]. Moreover, a decline in regulatory B cells (Bregs) results in the diminished production of anti-inflammatory mediators like IL-10, worsening immune system dysfunction.

The pathogenic network extends to macrophage interactions: B cell-derived IL-6 and IFN-γ polarize macrophages toward the proinflammatory M1 phenotype, while defective Breg-mediated IL-10 production further amplifies macrophage activation. M1 macrophages recognize B cell-generated immune complexes via Fcγ receptors, triggering complement activation and releasing TNF-α and IL-1β, which directly damage podocytes [16]. The CD40-CD40L axis and macrophage-derived B cell activating factor (BAFF) form a positive feedback loop that sustains B cell survival and autoantibody production [17]. Rituximab ameliorates disease by depleting B cells, thereby reducing autoantibody production, indirectly suppressing T cell hyperactivation, restoring Treg/Th17 balance, and modulating macrophage polarization. This integrated B cell–T cell–macrophage interplay constitutes the core immunopathogenic mechanism in INS, offering novel therapeutic targets for intervention [18].

(3) Sustaining immune homeostasis

Recent studies have significantly advanced our understanding of Bregs in maintaining immune tolerance, as well as their potential dysregulation in NS. Morath C et al. demonstrated that donor-derived modified immune cells, specifically peripheral blood mononuclear cells, when administered prior to kidney transplantation, acquired immunosuppressive properties following short-term treatment. This induced specific immunosuppression against allogeneic donors and was significantly associated with an increase in regulatory B lymphocytes [19]. Yang B et al. demonstrated that, in NS, alterations in the function or number of Bregs may disrupt immune tolerance, leading to an increased activation of inflammatory pathways. This includes the activation of Th17 cells and T helper 1 cells, as well as the production of pathogenic antibodies. This triad of immunological disturbances creates a permissive environment for glomerular injury, particularly through complement activation and Fc receptor-mediated inflammation. The resulting immune imbalance can exacerbate glomerular damage [20]. The study further demonstrated that the severity of proteinuria correlated inversely with circulating Breg frequencies, suggesting their potential role as both biomarkers and therapeutic targets.

Emerging evidence suggests that the pivotal role of B cell-derived cytokines in the pathogenesis of NS extends beyond their conventional immune functions. Activated B cell populations, particularly transitional B cells and memory B cells, along with their terminally differentiated plasma cell counterparts, constitute significant sources of proinflammatory mediators that orchestrate glomerular injury. These cells secrete IL-6, which drives Th17 polarization while simultaneously suppressing Treg development, thereby disrupting immune homeostasis. Furthermore, B cell-derived TNF-α promotes endothelial activation and induces the expression of adhesion molecules on podocytes, contributing to filtration barrier dysfunction. The autocrine production of BAFF by these cells perpetuates the survival of autoreactive B cell clones, establishing a self-sustaining inflammatory cascade. This pathogenic cytokine network, characterized by persistent IL-6, TNF-α, and BAFF signaling, not only exacerbates podocyte injury but also underlies the therapeutic resistance observed in SRNS cases, providing a compelling rationale for targeted B cell depletion strategies [21].

(4) Promote glomerulosclerosis

B cells play a role in the progression of glomerular sclerosis [22], a pathological condition characterized by the hardening and scarring of the glomeruli [23]. This sclerosis serves as a critical contributor to the decline of kidney function, impairing the glomeruli’s ability to efficiently filter blood. Chronic kidney disease can progress to end-stage renal disease, a critical condition characterized by a glomerular filtration rate below 15 mL/(min. 1.73 m^2^), which often necessitates dialysis or kidney transplantation.

In essence, the role of B cells in the pathogenesis of NS is both significant and multifaceted. B cells, which are pivotal in humoral immunity, contribute to the production of harmful autoantibodies, exacerbate inflammatory responses, and facilitate the progression to glomerular sclerosis (Figure 2). A thorough comprehension of these mechanisms is essential for developing targeted therapeutic strategies that can alleviate the influence of B cells and enhance the prognosis for patients facing this formidable condition.

## 3. Innovative Therapies Targeting B Cells

(1) Rituximab

Rituximab, a type I anti-CD20 antibody, eliminates B cells via mechanisms including antibody-dependent cellular cytotoxicity (ADCC), complement-dependent cytotoxicity (CDC), as well as apoptosis. In SDNS and focal segmental glomerulosclerosis (FSGS), it effectively reduces relapse rates and steroid dependence by specifically targeting CD20+ B cells [24]. By depleting B cells, rituximab potentially disrupts the autoimmune processes believed to underlie certain forms of NS [25]. This depletion entails not only a decrease in cell numbers but also a substantial transformation of the immune microenvironment [26].

Rituximab has exhibited efficacy in the treatment of patients diagnosed with frequently relapsing NS (FRNS) and SDNS. A multicenter controlled trial, conducted across nine centers in Japan, has evidenced that rituximab effectively extends the duration of the relapse-free period among children afflicted with FRNS or SDNS. The study encompassed 52 patients, all over the age of two, who were randomly allocated to receive either rituximab (administered at a dosage of 375 mg/m^2^ weekly for a span of four weeks) or a placebo. The findings indicated that the median recurrence-free survival time was substantially prolonged in the rituximab cohort in contrast to the placebo cohort, with no marked disparity in the incidence of severe adverse events between the two groups [27]. Another multicenter clinical trial was conducted to assess the efficacy of rituximab in the treatment of FRNS/SDNS. The findings indicated that all 30 patients who were administered 1–2 doses of rituximab remained in remission throughout the one-year follow-up period. The annual relapse rate experienced a significant reduction, decreasing from 88 to 22 episodes. Additionally, there was a substantial decrease in both the maintenance dose and the cumulative dosage of steroids [28]. Several clinical trials have demonstrated that rituximab exhibited greater efficacy compared to low-dose mycophenolate mofetil, tacrolimus, and oral cyclophosphamide [29,30,31]. With regard to the long-term safety of medication, children diagnosed with FRNS or SDNS who undergo repeated rituximab therapy exhibit sustained clinical symptom improvement. Although side effects are deemed tolerable, they may precipitate severe complications. These research findings lend support to the practice of re-administering rituximab [32]. An international retrospective study conducted across fifteen countries has revealed that maintenance therapy with rituximab is correlated with prolonged relapse-free survival, surpassing three years, in adults diagnosed with podocytopathies [33]. A meta-analysis encompassing 21 studies involving 382 adult patients with MCD/FSGS indicated that rituximab could serve as an effective and relatively safe alternative to CNIs or prednisone for the majority of adult patients with FRNS or SDNS [34]. The effectiveness and safety of rituximab in treating childhood steroid-resistant NS (SRNS) remain indeterminate. An international retrospective cohort study assessed the efficacy of rituximab in 246 children diagnosed with SRNS from twenty-eight centers spanning four continents. The study elucidates the therapeutic potential of rituximab in pediatric SRNS, particularly when administered early in the treatment regimen, with complete remission emerging as a robust indicator of sustained renal function [35]. MRNS is correlated with an unfavorable renal prognosis. A multicenter, single-arm clinical trial demonstrated that six pediatric patients with MRNS who were administered four doses of 375 mg/m^2^ rituximab, in conjunction with baseline cyclosporine and steroid pulse therapy, effectively diminished the urinary protein/creatinine ratio [36].

Additionally, rituximab has demonstrated an impact on B cell-derived cytokines and autoantibody production, both of which can contribute to glomerular damage. Despite its successes, several limitations are notable: (1) Variable Efficacy: The clinical effectiveness of rituximab in treating refractory SRNS patients is suboptimal. A study conducted by Roccatello D et al. revealed that merely a small fraction (1 out of 8) of adult patients diagnosed with FSGS exhibited beneficial outcomes in response to high doses of rituximab [37]. A study investigated the therapeutic interventions for recurrent FSGS following renal transplantation. The administration of rituximab yielded inconsistent outcomes, as documented in reports indicating remission rates ranging between 57% and 100% [38]. Several studies have suggested that FSGS is associated with an increased probability of non-response [39,40,41]. (2) Immunogenicity: The development of anti-rituximab antibodies occurs in 10–20% of patients subsequent to repeated administrations, leading to secondary non-response [4,42]. Antibodies targeting rituximab (ARA) are linked to adverse occurrences and therapeutic efficacy in children suffering from complex SDNS. The research identified the presence of ARA in 5 out of 13 participants. Those with a positive ARA status experienced a quicker recovery time for CD19 and significantly shorter periods without recurrence compared to those with a negative ARA status. Moreover, individuals with a positive ARA status were at a greater risk for severe infusion reactions and serum sickness [42]. Boyer-Suavet et al. suggest that the presence of ARA impacts the therapeutic efficacy in MN. Individuals with a high recurrence rate should consider alternative therapeutic interventions. Humanized anti-CD20 antibodies could represent a viable alternative for those afflicted with ARA [43]. (3) Incomplete Depletion: Tissue-resident and memory B cells frequently persist, thereby contributing to relapse [44]. Tur C et al. conducted a series of continuous lymph node biopsies on five patients prior to and following CD19-CAR T cell therapy, as well as on five patients subsequent to rituximab therapy. The CD19-CAR T cell therapy resulted in the complete depletion of CD19+ and CD20+ B cells within the lymph nodes, a feat not achieved by rituximab treatment. Post-CD19-CAR T cell therapy, the structural integrity of the follicles was compromised, and follicular dendritic cells were depleted within the lymph nodes; such alterations were not observed following rituximab treatment [45]. Reddy VR et al. found that in all patients with poor response to rituximab therapy, immunohistochemical analysis revealed persistent intraglomerular B cell infiltration despite undetectable circulating B cell populations in the peripheral blood. Comparative therapeutic assessment demonstrated obinutuzumab’s enhanced efficacy over rituximab in achieving complete B cell depletion [46]. (4) Safety Considerations: A retrospective cohort study was executed across sixteen pediatric nephrology centers situated in ten countries, encompassing children who had undergone two or more administrations of rituximab for the treatment of FRNS or SDNS. The findings revealed that hypogammaglobulinemia (50.9%) was the most prevalent condition, albeit only 22% of these instances were clinically significant. Infections (4.5%) and neutropenia (3.7%) were infrequent and manageable. Noteworthy risk factors for hypogammaglobulinemia comprised a younger age at the time of treatment and steroid resistance [32]. A retrospective investigation conducted in China revealed that 64.0% of 487 adult patients administered rituximab encountered adverse events (AE), with the majority being infectious in nature (40.6%) and a smaller proportion consisting of non-infectious events (23.4%) [47]. An additional safety concern associated with rituximab pertains to the incidence of infusion-related reactions (IRR), particularly during the initial infusion phase. The clinical manifestations of infusion reactions induced by rituximab generally emerge within minutes to a span of 120 min subsequent to the commencement of the infusion [48]. Mild symptoms encompass fever, chills, rash, and headache, whereas severe instances may pose a threat to life [49].

All of these factors have spurred the investigation into innovative anti-CD20 molecules [4,42].

(2) Ofatumumab

Ofatumumab, initially developed for hematologic malignancies and approved in 2020 for multiple sclerosis (MS), has demonstrated superior efficacy over existing therapies in phase III clinical trials, significantly reducing annual relapse rates by 50.5% to 58.8% and delaying disability progression in patients with relapsing MS. This humanized CD20 antibody offers a convenient, once-monthly subcutaneous injection option, potentially becoming the first B cell therapy that can be self-administered at home. It binds to a unique epitope on the CD20 antigen, inducing B cell lysis through CDC and ADCC. By selectively depleting CD20+ B cells, ofatumumab disrupts inflammatory processes involved in pathogenesis, such as autoantibody production and T cell activation. Unlike some anti-CD20 therapies, its subcutaneous administration facilitates at-home dosing, thereby improving patient accessibility [50].

Basu B. endeavored to utilize ofatumumab for the treatment of patients with rituximab-resistant SRNS, achieving a significant effect [51]. The initial report of successful therapy for post-transplant recurrent FSGS with ofatumumab was documented by Wang CS et al. For individuals with hypersensitivity to ofatumumab, a desensitization protocol can be utilized [52]. Ofatumumab could potentially serve as an efficacious and secure therapeutic option for individuals diagnosed with MN who exhibit intolerance to rituximab [53]. The fully human anti-CD20 monoclonal antibody ofatumumab features an elongated binding domain and demonstrates a heightened affinity for CD20 in comparison to rituximab, which may translate into enhanced therapeutic efficacy for individuals. Nevertheless, the research conducted by Bernard J et al. indicates that ofatumumab is ineffective in the treatment of NS recurrence subsequent to kidney transplantation [54]. Ofatumumab could represent a viable treatment option for patients with refractory childhood NS.

However, in a monocentric randomized clinical investigation assessing long-term therapeutic outcomes, ofatumumab monotherapy failed to demonstrate superiority over rituximab in maintaining disease remission for pediatric NS patients with a dual dependency on steroids and CNIs [55]. Research by Colucci et al. demonstrated that among pediatric FRNS/SDNS cases receiving B cell depletion therapy, both a younger age at treatment initiation and pre-treatment memory B cell levels served as independent predictors for earlier disease recurrence and faster immune cell repopulation [56].

According to the SDNS study, the standard dosage (1500 mg/1.73 m^2^) exhibited efficacy that was on par with rituximab. The study’s outcomes do not corroborate any purported benefits of administering ofatumumab at the standard low dose (1500 mg/1.73 m^2^) to children suffering from MRNS [57]. High-dose regimens, comprising 300 mg/1.73 m^2^ followed by 2000 mg/1.73 m^2^ administered five times, resulted in remission in 80% of cases involving rituximab-resistant minimal residual disease neuroblastoma; however, safety considerations, such as respiratory reactions, remain [51]. New prospective randomized clinical trials of ofatumumab in MRNS should be conducted with consideration given to higher doses. Research into the adverse reactions of ofatumumab has also been undertaken. Pediatric patients with SDNS demonstrated excellent tolerance to infusion therapy, with minimal acute reactions. Mild hypersensitivity manifestations (cutaneous eruptions, respiratory symptoms, and pyrexia) occurred in 5% of rituximab versus 18% of ofatumumab recipients, all effectively managed with prophylactic salbutamol. Intermediate-term adverse events (arthropathy and pulmonary complications) showed higher incidence with rituximab (2% and 1%, respectively). Late-onset infectious complications (3–9 months post-treatment) exhibited a comparable frequency between groups and responded appropriately to antimicrobial interventions [58].

(3) Obinutuzumab

Obinutuzumab is a monoclonal antibody utilized in the management of specific B cell malignancies. It functions by targeting the CD20 protein located on the surface of B cells, thereby instigating their elimination. Differentiating from earlier anti-CD20 antibodies, Obinutuzumab is classified as a type II anti-CD20 monoclonal antibody, engineered to optimize its ADCC. This pharmacological optimization confers superior B cell depletion efficacy when compared to conventional type I anti-CD20 monoclonal antibodies, including rituximab, ocrelizumab, and ofatumumab. Marinov AD et al. conducted a study on lupus-prone MRL/lpr mice, which expressed human CD20 on their B cells. These mice were administered either rituximab or obinutuzumab, and the subsequent B cell depletion was assessed under various conditions. Although both anti-CD20 antibodies alleviated early stages of the disease, obinutuzumab demonstrated superior efficacy in key parameters. Additionally, obinutuzumab exhibited advantageous effects in a model representing advanced stages of the disease [59]. Rituximab has served as a fundamental treatment for many years; however, obinutuzumab has been specifically developed to surmount resistance mechanisms, providing increased direct cell death and ADCC. Clinical trials have demonstrated the efficacy of obinutuzumab in the treatment of previously untreated chronic lymphocytic leukemia (CLL) and follicular lymphoma, notably in attaining more profound responses and diminishing the incidence of early progression [60].

Obinutuzumab has demonstrated considerable efficacy and safety in the treatment of adult patients with MCD and FSGS who experience frequent relapses or fail to attain remission. A retrospective analysis conducted by Lin Y et al. examined 11 adult patients diagnosed with MCD or FSGS who had undergone multi-target therapy, including rituximab, and continued to experience frequent relapses or were unable to achieve remission. The median time to the first relapse-free period was 12.1 months, during which two patients with FSGS experienced relapses, while the rest of the patients remained in remission until the conclusion of the follow-up period. Adverse reactions were predominantly mild and included infusion reactions, infections, somnolence, and hypogammaglobulinemia [61]. The report by Dossier C et al. examines the application of obinutuzumab in pediatric patients diagnosed with SDNS or FRNS, who exhibited resistance to or experienced relapse subsequent to rituximab therapy. The study’s outcomes indicate that B cell depletion was accomplished in all subjects, with a median duration of 8.3 months, which is notably more protracted than the effects observed with rituximab [62]. Patients who underwent treatment with obinutuzumab subsequent to relapse following rituximab therapy experienced an extended duration of B cell depletion and exhibited a higher rate of relapse-free survival in comparison to those demonstrating resistance to rituximab. The safety profile of obinutuzumab was observed to be analogous to that of rituximab. Consequently, obinutuzumab might present as a viable alternative for pediatric patients with renal impairment who are unable to tolerate rituximab [63,64,65]. Large-scale, randomized controlled trials are necessary to further substantiate its efficacy and safety in comparison with rituximab and alternative treatments.

(4) Ublituximab

Ublituximab is a novel monoclonal antibody engineered to specifically target the CD20 antigen. It binds to a unique epitope on the CD20 molecule, which is distinct from those recognized by rituximab, ofatumumab, and obinutuzumab. This chimeric anti-CD20 antibody is characterized by its heightened affinity for FcγRIIIa, thereby facilitating robust ADCC [66]. Ublituximab has demonstrated efficacy in the treatment of relapsing forms of MS. Through the depletion of B cells, ublituximab aids in the reduction of inflammatory processes that contribute to the demyelination of nerve fibers [66,67,68]. In hematologic malignancies, including CLL, the capacity of ublituximab to target CD20-positive B cells can result in a diminution of tumor burden and enhance patient outcomes [69]. Initial investigations within the field of hematology indicate promise, yet data specific to nephrology are scant. The B cell depleting mechanism of action of ublituximab may assist in alleviating the autoimmune response, with the potential to preserve renal function and diminish proteinuria.

## 4. Targeting Plasma Cells

CD38 is a transmembrane glycoprotein expressed on myeloma cells, as well as to a lesser extent on normal lymphoid cells, myeloid cells, and certain non-hematopoietic tissues. CD38 antibodies target immune cells expressing the CD38 glycoprotein, leveraging mechanisms like CDC, ADCC, and antibody-dependent cellular phagocytosis (ADCP) to eliminate pathogenic plasma cells and modulate immune responses [70,71]. Daratumumab is a CD38 monoclonal antibody that demonstrates a substantial single-agent response rate in the treatment of multiple myeloma [68]. It targets CD38-expressing myeloma cells via immune-mediated mechanisms (CDC, ADCC, ADCP) and direct apoptosis, while also influencing CD38-positive immune cells such as Bregs, myeloid-derived suppressor cells, and a recently identified immunosuppressive CD38+ Treg subset. These immunomodulatory effects imply additional mechanisms of action for daratumumab beyond the direct elimination of tumor cells [70,72]. Beyond these immune-mediated mechanisms, daratumumab demonstrates anti-tumor activity through interaction with macrophages via the Fc-FcγR pathway. It stimulates macrophage activation in murine models and facilitates ADCP of malignant cells through Fc-FcγR interaction in vitro [73]. Nonetheless, its potential role in the context of renal diseases, particularly those associated with plasma cell dyscrasias, has garnered considerable attention due to its mechanism of action and immunomodulatory properties.

For individuals who have received renal transplantation, to mitigate the incidence of acute rejection episodes, immunosuppressive therapy is generally mandated. Antibody-mediated rejection (AMR) in kidney transplant recipients represents a primary cause of graft failure. Targeting plasma cells and natural killer (NK) cells through CD38-directed therapies may serve as a promising strategy to mitigate AMR. Doberer K et al. reported the application of the CD38 monoclonal antibody daratumumab in kidney transplant recipients. Daratumumab effectively depleted CD138+ plasma cells in the bone marrow and blood. It also substantially reduced NK cell counts in both blood and graft tissue. Daratumumab’s dual depletion of these cells likely contributed to AMR resolution [74]. For patients with MN who exhibit an inadequate response to conventional therapies, treatment with CD38 monoclonal antibodies may also be considered as a viable option. The M-PLACE trial (NCT04145440), a phase 1b/2a open-label investigation, evaluated the fully humanized anti-CD38 mAb felzartamab in anti-PLA2R antibody-positive primary MN patients with high-risk features. The treatment resulted in a serological response (76.9% of participants) with rapid anti-PLA2R antibody titer reduction. Additionally, some patients exhibited a partial improvement in proteinuria and serum albumin levels, while the treatment was associated with good safety profiles [75].

Lupus nephritis (LN) constitutes the most prevalent severe complication associated with systemic lupus erythematosus (SLE). In instances where lupus is active, the production of excessive autoantibodies can occur, resulting in tissue damage [76,77]. The employment of a CD38 monoclonal antibody to eliminate activated plasma cells presents as a potentially efficacious therapeutic strategy. Roccatello D et al. conducted an assessment of daratumumab in six individuals diagnosed with treatment-resistant LN. Following 12 months of treatment, five out of the six patients exhibited marked clinical amelioration, evidenced by diminished disease activity, reduced proteinuria, and lower serum creatinine levels. Serological enhancements encompassed the seroconversion of anti-dsDNA antibodies, a decrease in inflammatory markers, and elevated levels of complement C4 and IL-10. However, one patient did not respond positively after a period of six months. These results imply that daratumumab monotherapy could be a prospective treatment for patients with refractory LN [78]. Chiarenza DS et al. described two cases of adolescents with refractory LN/SLE who were successfully treated with a combination of a single infusion of rituximab and daratumumab, demonstrating safety and efficacy [79]. While CD38 monoclonal antibodies have demonstrated potential in alleviating symptoms among patients with LN, the necessity for the additional substantiation of their safety and efficacy in this patient population persists. This validation should be pursued through the implementation of multicenter randomized controlled trials.

The capacity of CD38 monoclonal antibodies to deplete plasma cells is transient. To achieve a sustained inhibition of the differentiation of autoreactive B cell precursors into autoreactive plasma cells, it is imperative that these antibodies be administered in conjunction with other immunosuppressive agents [80]. In the domain of NS, emphasis is placed on clinical trials that incorporate the combination of anti-CD20 and anti-CD38 therapies. A Phase 2 proof-of-concept study (NCT05704400, DUAL-1) has been proposed to evaluate the efficacy of the combination of rituximab and daratumumab in sustaining drug-free remission among children and young adults diagnosed with SRNS and those experiencing recurrence of NS post-transplantation. The study concentrates on the concurrent administration of rituximab and daratumumab for the management of SRNS in pediatric and young adult populations, suggesting that targeting both B cells and plasma cells could be more efficacious than rituximab monotherapy in treating complex cases of NS. The research indicates that a reduction in IgM levels and CD38+ plasma cells is associated with disease remission, thereby suggesting a potential pathological involvement of these cells and antibodies [81]. The research indicates that daratumumab may contribute to the maintenance of remission following B cell recovery, potentially through its action against long-lived plasma cells. The study conducted by Dossier C et al. demonstrated that the combination of obinutuzumab and daratumumab was efficacious in achieving prolonged B cell depletion and remission in patients with SDNS who are challenging to treat [82]. The combination therapy was generally tolerable, with manageable adverse effects dominated by infusion reactions, cytopenias, and immunoglobulin depletion requiring IVIG support. Vigilance for infections and long-term immune monitoring remain essential [81,82]. Employing agents that target both CD20 and CD38 may represent a more efficacious therapeutic approach for the treatment of SRNS. This dual targeting strategy aims to surmount the constraints inherent in existing treatments and to offer a holistic method for B cell depletion. It is imperative that additional randomized controlled trials be conducted to substantiate the effectiveness of this approach and to elucidate the distinct contributions of each monoclonal antibody in sustaining remission.

The principal adverse effects linked to CD38 monoclonal antibodies include IRR (such as pyrexia, chills, and dyspnea), hematologic toxicities (including neutropenia, anemia, and hypogammaglobulinemia), and an elevated risk of infections. Additionally, these agents may provoke immune-mediated effects, such as T cell activation, which can result in autoimmune phenomena or graft-versus-host disease. Renal transplant recipients are particularly susceptible to acute rejection. Prolonged administration of these agents necessitates meticulous monitoring for secondary malignancies and cardiovascular events. Prophylactic strategies, encompassing corticosteroids and antihistamines, are advised to reduce risks. Regular monitoring of complete blood counts and IgG levels is recommended, with the provision of intravenous immunoglobulin or granulocyte colony-stimulating factor as supportive therapy when required. Although generally manageable, AEs necessitate individualized risk assessments, particularly in immunocompromised patients or transplant recipients, mandating vigilant surveillance.

## 5. CAR T Cells

CAR T cell therapy, a groundbreaking immunotherapy initially developed for the treatment of cancer, entails the genetic modification of a patient’s T cells to recognize and attack specific antigens [83,84,85]. CAR T cells are engineered by genetically altering a patient’s T lymphocytes to express a synthetic receptor composed of the following components: (1) An extracellular domain, which is a single-chain variable fragment (scFv) derived from monoclonal antibodies, facilitating the recognition of antigens such as CD19 or BCMA. (2) A hinge/spacer segment, which provides the necessary flexibility and ensures optimal antigen binding, derived from sequences such as those of CD8 or IgG4. (3) A transmembrane domain, which secures the CAR to the T cell membrane, sourced from molecules like CD28 or CD3ζ. (4) Intracellular signaling domains, which are responsible for the activation of T cell effector functions [85,86]. The CAR T cell therapy encompasses the extraction of autologous T cells via leukocyte isolation, the insertion of CAR genes employing viral vectors, such as lentiviruses, or non-viral methodologies, including CRISPR and transposons, the ex vivo expansion of the genetically modified T cells to attain therapeutic quantities, and the subsequent re-administration of these CAR T cells to the patient [87]. Upon their introduction into the patient, CAR T cells detect antigens on the target cells using the scFv, proliferate, and destroy the target cells by releasing perforin/granzyme or through the Fas/FasL pathways. Additionally, the enduring CAR T cells ensure continuous immune monitoring [88]. Recently, its potential has expanded into autoimmune and inflammatory diseases, including kidney disorders.

Self-reactive B lymphocytes are integral to the pathogenesis of autoimmune disorders, including SLE, rheumatoid arthritis (RA), and MS. Monoclonal antibody therapies designed to deplete B cells, such as rituximab, have demonstrated limited efficacy due to the persistence of self-reactive B cells within lymphoid organs and sites of inflammation. Consequently, CAR-T cell therapy has been proposed as a therapeutic approach for autoimmune diseases [89].

Given the initially promising efficacy of CAR-T cell therapy in the context of autoimmune diseases, the traditional treatment paradigms for immune-mediated kidney diseases are evolving towards more targeted B cell therapies. Empirical evidence indicates that autoantibodies and autoreactive B cells are the principal pathogenic mechanisms underlying glomerulonephritis. B cells are instrumental not only in the production of antibodies but also in the activation of the complement system and in mediating renal cell damage through antigen presentation, co-stimulatory signaling, and cytokine-regulated pathways [6,7,8,9]. Considering that CD19 is exclusively expressed on B lymphocytes, the utilization of cytotoxic CAR cells endowed with CD19-targeting capabilities presents a novel therapeutic approach for immune-mediated kidney diseases. Contemporary investigations have ascertained that autoimmune podocytopathies are distinguished by the existence of podocyte autoantibodies, frequently resulting in INS among pediatric populations. Research has demonstrated that a considerable proportion of INS patients manifest these autoantibodies, which are associated with the severity of the disease [8,90]. This subgroup indicates an autoimmune reaction that impacts the functionality of podocytes, underscoring the significance of identifying these antibodies to facilitate targeted therapeutic interventions and enhance patient prognoses [91]. At present, there is no effective treatment for multi-drug-resistant steroid-resistant NS (MDR-SRNS), which has a high risk of progression to kidney failure, which is in urgent need of new treatment methods. Mao J et al. are currently conducting two Phase 1 clinical trials (NCT06842589, NCT06553898) to assess the effectiveness and safety of CAR-T cells in the treatment of patients with MDR-SRNS. The studies were designed with three dosage groups (0.3 × 10^^5^/kg, 1 × 10^^5^/kg, 3 × 10^^5^/kg), starting from the low-dose group and increasing stepwise to explore the safe and effective dose. Nonetheless, surmounting challenges related to toxicity, cost, and resistance are imperative. As advancements in the design and delivery of CARs continue, this therapeutic modality may expand its application to a broader spectrum of indications in the near future.

## 6. Others

In addition to antibodies, other biological agents are currently being investigated in the field of NS.

BAFF and APRIL (a proliferation-inducing ligand) are key factors regulating B cell survival, differentiation, and antibody secretion. Their over-expression has been implicated in the pathogenesis of multiple autoimmune diseases. Telitacicept, a novel fully human recombinant TACI-Fc fusion protein, exhibits a dual-targeting capability by binding both BAFF and APRIL, thereby competitively inhibiting their interactions with receptors (BCMA, TACI, and BAFF-R). This mechanism suppresses B cell maturation and plasma cell differentiation, ultimately reducing autoantibody production [92]. Notably, telitacicept demonstrates potential efficacy against long-lived plasma cells, a cell population resistant to conventional immunosuppressive therapies.

Clinical trials have validated its therapeutic potential. In Phase IIb/III studies involving SLE patients, the subcutaneous administration of telitacicept (80–240 mg weekly) significantly improved the SLE Responder Index (SRI-4) and reduced disease activity (SLEDAI scores), with a favorable safety profile [93]. Similarly, a Phase II trial in IgA nephropathy (IgAN) reported a 49% reduction in 24 h urinary protein excretion at the 240 mg dose, prompting FDA approval for Phase III investigation [94]. Compared to monoclonal antibodies targeting single pathways, such as belimumab (anti-BAFF) or rituximab (anti-CD20), telitacicept’s dual inhibition of BAFF/APRIL may offer superior clinical efficacy. Importantly, its activity against CD20− long-lived plasma cells suggests therapeutic promise in refractory nephropathies. Supporting evidence includes case reports documenting sustained remission in MN patients following telitacicept therapy [95,96]. Mechanistically, Li et al. elucidated that IFN-γ-mediated BAFF pathway activation drives aberrant B cell responses and autoantibody production in steroid-sensitive NS [97]. These findings position telitacicept as a rational targeted therapy for NS, particularly in cases with dysregulated IFN-γ/BAFF signaling. Clinical trial of telitacicept for children with FRNS/SDNS are also ongoing (NCT06125405). Further studies are warranted to confirm its long-term efficacy and safety in NS.

Bispecific T cell Engagers (BiTEs) represent a promising immunotherapeutic approach by leveraging T cell-mediated cytotoxicity to target pathogenic B cells. These engineered molecules consist of two scFvs: one binds CD3 on T cells to induce activation, while the other targets B cell surface antigens (e.g., CD19 or BCMA), redirecting T cells to eliminate autoreactive B cells. Unlike conventional monoclonal antibodies (e.g., rituximab), which rely on ADCC or CDC, BiTEs achieve deeper and more sustained B cell depletion by harnessing the potent cytotoxic machinery of T cells, including perforin/granzyme release and cytokine-mediated killing [98,99]. This mechanism enhances tissue penetration, particularly in lymphoid organs where residual B cells often evade monoclonal antibody therapies. Preclinical and early clinical data suggest that BiTEs targeting CD19 (e.g., blinatumomab) or BCMA (e.g., teclistamab) may overcome the limitations of current therapies by depleting broader B cell subsets, including plasmablasts and some plasma cells, thereby reducing autoantibody production [99,100]. Perico L et al. developed bispecific autoantigen-T cell engagers that precisely eliminate autoreactive B cells by linking disease-specific autoantigens (e.g., PLA2R in MN) to T cell-activating anti-CD3 antibodies [101]. This approach represents a potential off-the-shelf, antigen-specific therapy that could replace broad immunosuppression for autoantibody-mediated diseases. However, challenges such as cytokine release syndrome (CRS), immune effector cell-associated neurotoxicity syndrome, T cell exhaustion, and infection risks due to prolonged B cell aplasia necessitate careful optimization. A clinical trial is currently underway to evaluate the safety and effectiveness of blinatumomabin treating children with CNI-resistant or intolerant SRNS (NCT06607991). Future directions include engineering BiTEs with improved safety profiles (e.g., CRS-mitigating designs), exploring combination strategies with BAFF inhibition, and developing long-acting formulations. While further clinical validation is required, BiTEs hold significant potential as a precision therapy for refractory NS, offering a novel paradigm to disrupt B cell-driven autoimmunity through T cell engagement.

## 7. Challenges and Perspectives

**Figure 3:**
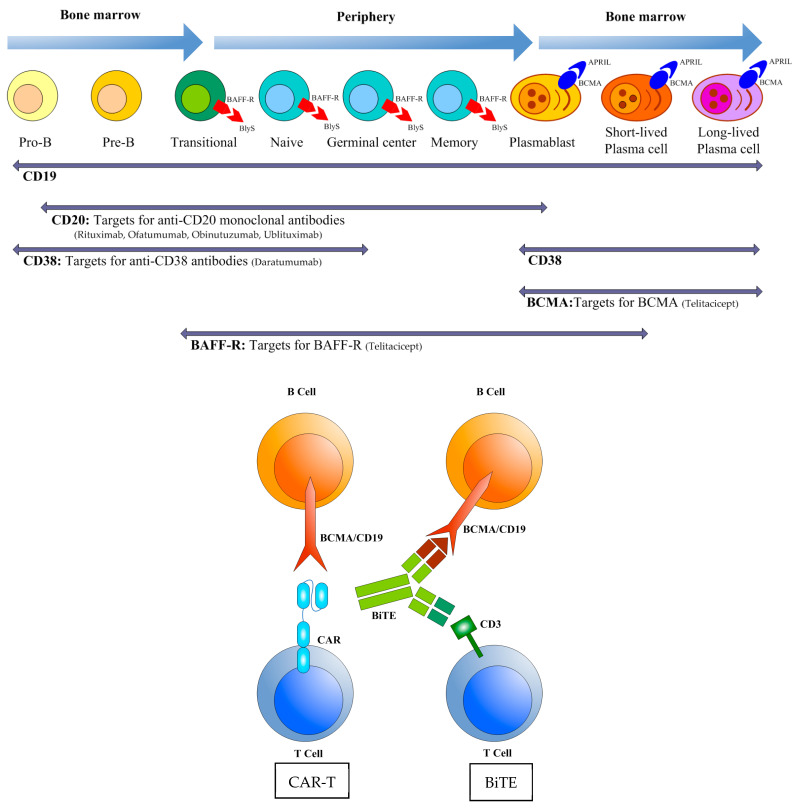
Dynamic alterations in surface marker expression throughout B cell development and prospective therapeutic targets (monoclonal antibody, CAR-T, BiTE).

Although B cell-targeted therapies have demonstrated significant potential in the treatment of NS (Figure 3), their widespread clinical application faces multiple challenges. Current anti-CD20 monoclonal antibodies, such as rituximab, fail to completely eliminate tissue-resident and memory B cells, contributing to disease relapse, with notable efficacy variations observed across different pathological subtypes (e.g., FSGS vs. MCD). Safety concerns remain prominent, including infection risks associated with B cell depletion (e.g., hypogammaglobulinemia), immunogenicity leading to anti-drug antibody formation, and CRS with CAR T cell therapies.

Furthermore, current challenges in the personalized treatment of NS include the lack of reliable biomarkers and significant disease heterogeneity. The inability to accurately predict patient responses to specific therapies (e.g., anti-CD20 or anti-CD38 antibodies) leads to empirical treatment selection (Table 2). For instance, FSGS patients exhibit significantly lower response rates to rituximab compared to MCD patients, though the underlying mechanisms remain unclear. The complex pathogenesis of NS involves diverse autoantibodies and immune pathways, necessitating subtype-specific therapeutic strategies (Table 3). Future research should focus on multi-omics approaches (e.g., single-cell sequencing, proteomics) to identify predictive biomarkers and advance precision medicine in NS management. The clinical translation of novel therapies for NS faces several challenges, including limitations in trial design, high costs, and regulatory hurdles. Most existing studies are small-scale, single-center, or retrospective (as shown in Table 4), with insufficient long-term follow-up data. For example, a CAR-T cell trial in NS (NCT06842589) enrolled only 9–18 patients and employed a single-arm design. Additionally, emerging therapies such as CAR-T cells and bispecific antibodies are prohibitively expensive, restricting accessibility in resource-limited settings; CAR-T therapy alone can cost hundreds of thousands of dollars and requires complex infrastructure. Regulatory and ethical concerns further complicate adoption, particularly in pediatric populations, where long-term safety data remain inadequate. Future efforts should prioritize multicenter randomized controlled trials, cost-effective alternatives (e.g., subcutaneous formulations), and international collaboration to accelerate clinical translation.

The limitations of monotherapy targeting either B cells or plasma cells may result in incomplete disease suppression. For instance, combined anti-CD20 and anti-CD38 antibody therapy (e.g., rituximab + daratumumab) has demonstrated synergistic effects in refractory NS, though the optimal regimen remains undetermined. A critical challenge lies in balancing immunosuppression, as excessive immune modulation may increase infection or malignancy risks. Future research should focus on optimizing sequential or combination strategies (e.g., B cell depletion followed by BAFF/APRIL inhibition) and developing immunomodulatory approaches, such as regulatory T cell expansion, to enhance efficacy while minimizing adverse effects (Figure 3).

## 8. Conclusions

Anti-B cell strategies have emerged as a promising therapeutic approach for managing NS, particularly in cases where conventional treatments fail. One of the primary targets in anti-B cell therapy is the depletion of B cells, which are crucial for the production of autoantibodies that contribute to glomerular damage. The anti-B cell arsenal in NS is expanding beyond rituximab. Next-generation anti-CD20 antibodies, plasma cell-targeted agents, and cellular therapies offer hope for refractory cases. Although anti-B cell strategies have revolutionized the treatment of NS, their clinical application still faces multiple challenges, including variable efficacy, safety concerns, lack of individualized approaches, and limited accessibility. Future research should prioritize elucidating underlying mechanisms, identifying predictive biomarkers, optimizing clinical trial designs, and reducing treatment costs to facilitate successful bench-to-bedside translation. Only through multidisciplinary collaboration and therapeutic innovation can we develop more effective and safer treatment options for NS patients.

## Figures and Tables

**Table 1:** Relevant therapeutic indications, approved regions, and research priorities.

Drugs	Indications	Approved Regions	Future Research
Rituximab	Adults with B cell malignancies (e.g., CLL,ALL) and autoimmune diseases (e.g., RA), pediatric CD20-positive ALL,MCD(FRNS/SDNS)	Worldwide, including the US (FDA), EU (EMA), and China (NMPA).	Focuses on personalized dosing; predictive biomarkers; combination therapies with novel agents; long-term safety monitoring.
Ofatumumab	Adult patients with relapsing MS and CLL, but no widely approved indications in children.	Worldwide, including the US (FDA), EU (EMA), and China (NMPA).	Expansion into pediatric populations; optimization of treatment protocols; long-term safety monitoring.
Obinutuzumab	Adult populations, for previously untreated CLL and follicular lymphoma, in combination with chemotherapy.	Worldwide, including the US (FDA), EU (EMA), and China (NMPA).	Potential in pediatric malignancies; subcutaneous formulation optimization; combination strategies with novel agents; long-term management of immune reconstitution and infection risks.
Blinatumomab	Pediatric and adult patients with relapsed or refractory B cell precursor ALL, and as consolidation therapy for patients with minimal residual disease-positive ALL.	Worldwide, including the US (FDA), EU (EMA), and China (NMPA).	Prioritize administration optimization; earlier-line efficacy evaluation; combination strategies with novel immunotherapies; and long-term safety monitoring.
Daratumumab	Adult patients with multiple myeloma. Pediatric applications remain investigational in relapsed/refractory acute leukemias.	Worldwide, including the US (FDA), EU (EMA), and China (NMPA).	Prioritize pediatric CD38+ malignancy trials; subcutaneous formulation development; novel immunotherapy combinations; enhancing management of adverse reactions.
CAR-T	Tisagenlecleucel is approved for patients up to 25 years of age with B cell precursor ALL that is refractory or in second or later relapse, while axicabtagene ciloleucel and brexucabtagene autoleucel are approved for adult patients with specific types of large B cell lymphoma and mantle cell lymphoma.	In major medical markets including the US(FDA), EU(EMA), Japan (PMDA), and China (NMPA), though specific approved indications may vary by region.	Expanding applications to solid tumors and earlier treatment lines; developing allogeneic “off-the-shelf” platforms to enhance accessibility; improving toxicity management for CRS and neurotoxicity; optimizing manufacturing and cost structure; establishing long-term safety and efficacy surveillance.
Telitacicept	Adult patients with SLE. While not yet approved for pediatric use, early clinical investigations are exploring its potential in childhood-onset autoimmune conditions.	Approved only in China (NMPA).	Pediatric autoimmune disease trials; expand indications for B cell mediated disorders; develop predictive biomarkers for treatment response; establish long-term immunologic monitoring protocols to assess infection risks.
Ublituximab	Adult patients with relapsing forms of MS. Not currently approved for pediatric use in any region.	Approval primarily in US(FDA)	Pediatric autoimmunity applications; dosing optimization; long-term monitoring of humoral immunity and infection risks; and exploration of combination strategies with novel immunomodulatory agents.

**Table 2:** Therapies in kidney diseases: comparative assessment.

Drug/Therapy	Mechanism	Safety	Durability of Response	Renal Research
Rituximab	Anti-CD20 mAb; depletes B lymphocytes	Infusion reactions (mild), hypogammaglobulinemia (long-term)	12–36 months; 30–40% relapse within 2 years (FRNS/SDNS)	[3,27,28,29,30,36,39,55,97,102,103,104,105,106]
Ofatumumab	2nd-gen anti-CD20 mAb; higher CD20 affinity, stronger B cell depletion	Fewer infusion reactions vs. rituximab; similar hypogammaglobulinemia risk	40% maintained efficacy within 2 years (rituximab-resistant MN) A low-dose single infusion was ineffective for MRNS	[30,55,57,107]
Obinutuzumab	Glycoengineered anti-CD20 mAb; enhanced ADCC	Grade 3–4 infections (15%); frequent infusion reactions	Achieving a 24 month relapse-free survival rate of 68% (FRNS/SDNS)	[62,63,64]
Daratumumab	Anti-CD38 mAb; targets plasma cells and activated B cells	Thrombocytopenia, infusion reactions, infection risk (respiratory)	Combined with obinutuzumab 60% 2 year relapse-free, median immunosuppressant free duration was 19.1 months (SDNS)	[74,75,79,81,82]
CAR T Cells	CD19/BCMA-targeted autologous T cells; eliminate pathogenic B/plasma cells	CRS, neurotoxicity, long-term B cell aplasia	No data	No data
Telitacicept	TACI-Fc fusion protein; inhibits BLyS/APRIL, blocks B cell differentiation	Upper respiratory infections, headache; no severe infusion reactions	Proteinuria IgAN: reduction sustained to 48 weeks	[94,95,96]
Blinatumomab	Bispecific CD19/CD3 mAb; redirects T cells to kill CD19+ B cells	CRS, neurotoxicity, cytopenias; rarely used outside oncology	No data	No data

**Table 3:** Comparative analysis of therapies for NS subtypes.

Subtype	Recommended Therapies
MCD	First-line: Rituximab (60–80% remission) Refractory (FRNS/SDNS/SRNS): Ofatumumab/Obinutuzumab/Rituximab + Daratumumab/MDR-SRNS: CAR-T may be considered as a potential therapeutic approach.
FSGS	First-line: Rituximab (30–50% remission) Refractory: Obinutuzumab/Ofatumumab may be considered as a potential therapeutic approach. Monoclonal gammopathy-related: Daratumumab/CAR-T may be considered as a potential therapeutic approach.
MN	Preferred: Obinutuzumab (60% remission, faster onset) or Rituximab (40–60%) Emerging: Telitacicept
IgAN	First-line: Telitacicept (34% improvement in proteinuria) Second-line: Rituximab

**Table 4:** Main clinical trials focused on anti-CD20 treatment for NS.

Drug Type	Clinical Trial	N	Study Design	Dose	Status	Phase	ClinicalTrials.gov ID
**Rituximab**	Rituximab for INS	30	RCT	375 mg/m^2^ (1 dose)	Completed	3	NCT04494438
	Efficacy and Safety of Rituximab to That of CNIs in Children With Steroid Dependent NS	120	RCT	375 mg/m^2^ (2–4 dose)	Completed	3	NCT02438982 [30]
	Ofatumumab Versus Rituximab in Children With Steroid and CNIs Dependent Idiopathic	140	RCT	375 mg/m^2^ (1 dose)	Completed	2	NCT02394119 [55]
	Rituximab in Multirelapsing MCD or FSGS (NEMO)	24	Single Group	375 mg/m^2^ (1 dose)	Completed	3	NCT00981838 [28]
	Efficacy and Safety of Rituximab in the First Episode of Pediatric Idiopathic	44	Single Group	375 mg/m^2^ (1 dose)	Completed	2	NCT04783675 [108]
	Compare Efficacy and Safety of Repeated Courses of Rituximab to That of Maintenance Mycophenolate Mofetil Following Single Course of Rituximab Among Children With Steroid Dependent NS (RITURNS II)	100	RCT	375 mg/m^2^ (3 dose)	Completed	3	NCT03899103 [109]
	Efficacy of Rituximab For the Treatment of CNIs Dependent NS During Childhood (NEPHRUTIX)	26	RCT	375 mg/m^2^ (2 dose)	Completed	1–2	NCT01268033 [12]
	Clinical Study of Rituximab for the Treatment for Idiopathic MN with NS (PRIME)	88	RCT	500 mg (2–4 dose)	Recruiting	3	NCT05914155
	Efficacy and Safety of Rituximab Versus Mycophenolate Mofetil in Children With SDNS	46	RCT	375 mg/m^2^ (2 dose)	Recruiting	2	NCT05843968
	Study of Rituximab Monotherapy on Children With New-onset NS: A Randomized Controlled Trial (STORM)	80	RCT	375 mg/m^2^ (4 dose)	Recruiting	3	NCT05734794
	Rituximab Plus Cyclosporine in Idiopathic MN	30	Single Group	1000 mg (6 dose)	Recruiting	2	NCT00977977
	Rituximab, Cyclophosphamide, and Corticosteroids in Primary MN	40	Single Group	375 mg/m^2^ (4 dose)	Recruiting	Not Applicable	NCT05679336
**Ofatumumab**	Ofatumumab Versus Rituximab in Children With Steroid and CNIs Dependent Idiopathic	140	RCT	1500 mg/1.73 m^2^ (1 dose)	Completed	2	NCT02394119 [55]
**Obinutuzumab**	Efficacy and Safety of Obinutuzumab Versus Rituximab in Childhood Steroid Dependant and Frequent Relapsing NS (OBIRINS)	88	RCT	300 mg/1.73 m^2^ (1 dose)	Recruiting	1–2	NCT05786768
**Daratumumab**	Efficacy of Anti-CD20 Ab Associated With Anti-CD38 in the Childhood Multidrug Dependent and Resistant NS	20	Single Group	16 mg /Kg (1 dose)	Recruiting	2	NCT05704400
**CAR T Cells**	Study of Therapeutic Efficacy of Anti-CD19 CAR-T Cell Therapy in Patients With MDR-SRNS	9–18	Single Group	Three dose groups (0.3 × 10^5^/kg, 1 × 10^5^/kg, 3 × 10^5^/kg)	Recruiting	1	NCT06842589
	Study of Therapeutic Efficacy of CAR-T Cell Therapy in Patients With MDR-SRNS	18	Single Group	Three dose groups (0.3 × 10^5^/kg, 1 × 10^5^/kg, 3 × 10^5^/kg)	Recruiting	1	NCT06553898
**Telitacicept**	Study of the Telitacicept in Pediatric Patients With Frequently Relapsing or Steroid Dependent NS (STERN)	20	Single Group	10–20 kg: 40 mg 20–40 kg: 80 mg 40–60 kg: 120 mg ≥60 kg: 160 mg	Recruiting	3	NCT06125405
**Blinatumomab**	Blinatumomab for CNI-Resistant/Intolerant SRNS in Children	6	Single Group	two 5 day cycles (5 µg/m^2^/day, maximum dose 9 µg/day)	Recruiting	1	NCT06607991

## Abbreviations

The following abbreviations are used in this manuscript:

ADCC	Antibody-dependent cellular cytotoxicity
ADCP	Antibody-dependent cellular phagocytosis
AE	Adverse events
AMR	Antibody-mediated rejection
ARA	Antibodies targeting rituximab
BAFF	B cell activating factor
BiTEs	Bispecific T cell Engagers
Bregs	Regulatory B cells
CAR	Chimeric antigen receptor
CDC	Complement-dependent cytotoxicity
CLL	Chronic lymphocytic leukemia
CNIs	Calcineurin inhibitors
CRS	Cytokine release syndrome
FRNS	Frequently relapsing NS
FSGS	Focal segmental glomerulosclerosis
IgAN	IgA nephropathy
IRR	Infusion-related reactions
LN	Lupus nephritis
MCD	Minimal change disease
MDR-SRNS	Multi-drug-resistant steroid-resistant NS
MN	Membranous nephropathy
MRNS	Multidrug-resistant NS
MS	Multiple sclerosis
NK	Natural killer
NS	Nephrotic syndrome
RA	Rheumatoid arthritis
scFv	Single-chain variable fragment
SDNS	Steroid-dependent NS
SLE	Systemic lupus erythematosus
SRNS	Steroid-resistant NS
TFH	Follicular helper T cells
